# Supplementary material for: The interactome of intact mitochondria by cross-linking mass spectrometry provides evidence for coexisting respiratory supercomplexes
Source: Mol Cell Proteomics. 2017 Dec 8;17(2):216–32. doi: 10.1074/mcp.RA117.000470 (PMC5795388; doi:10.1074/mcp.RA117.000470)
Supplement: Supplemental Data [file supp_RA117.000470_133922_0_supp_23978_fzffwf.zip › spectra_annotation/mito_spectra_annotation/39-2-15-1-4-1.pdf]

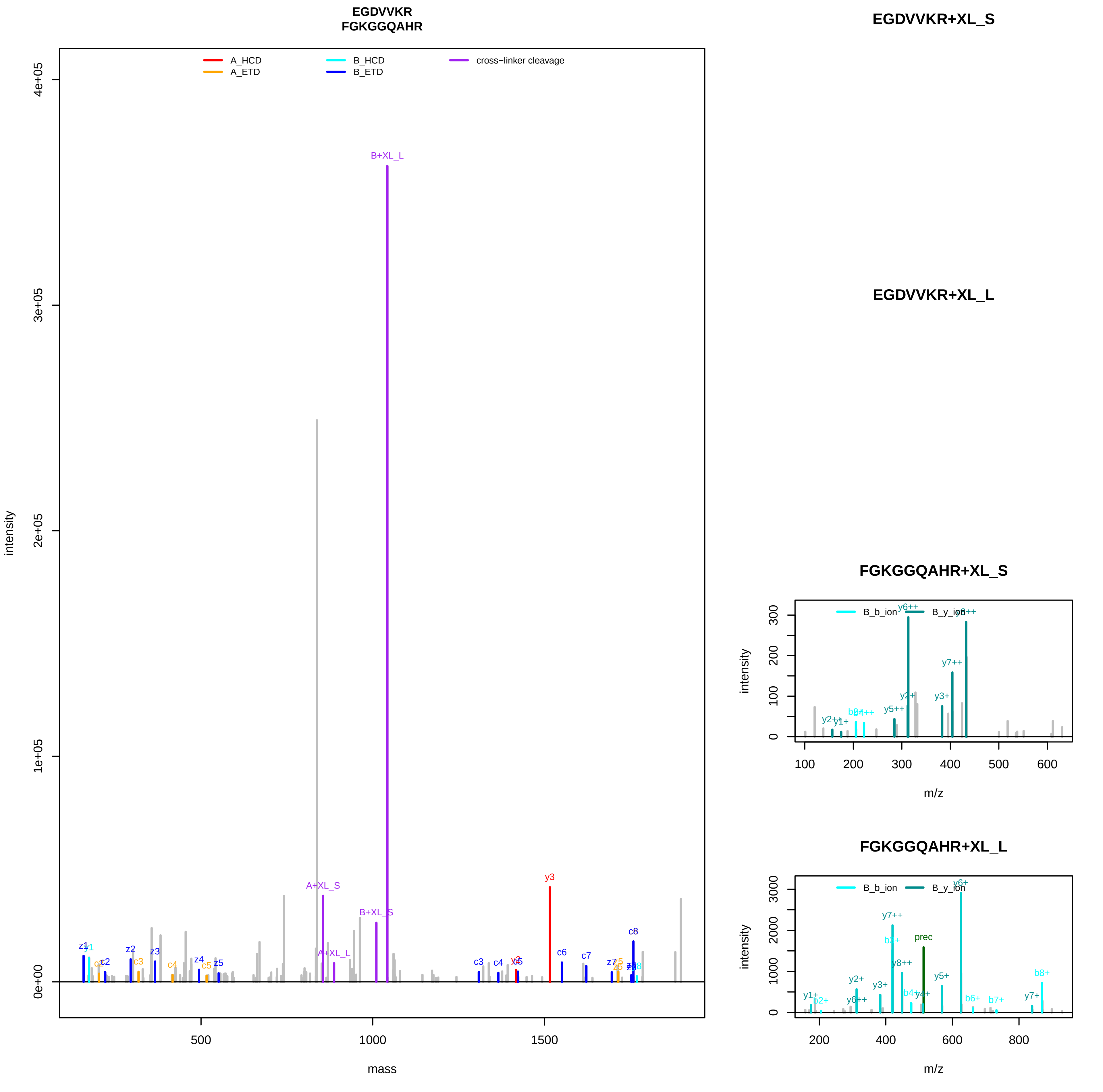

EGDVVKR
FGKGGQAHR
A_HCD
A_ETD
B_HCD
B_ETD
cross−linker cleavage
intensity
mass
0e+00
1e+05
2e+05
3e+05
4e+05
500
1000
1500
B+XL_L
A+XL_S
B+XL_S
A+XL_L
y3
z1
y1
c2
z2
c3
z3
c4
z4
c5
z5
c3
c4
c5
c6
c7
z7
c8
z8
EGDVVKR+XL_S
EGDVVKR+XL_L
FGKGGQAHR+XL_S
B_b_ion
B_y_ion
m/z
100
200
300
400
500
600
0
y6++
y7++
y3+
y5++
y2+
b4++
y1+
FGKGGQAHR+XL_L
1000
2000
3000
800
y6+
y7++
b3+
prec
y8++
y2+
y3+
y5+
y4+
b4+
y1+
b2+
b6+
b7+
y7+
b8+
